# M1-like macrophage contributes to chondrogenesis in vitro

**DOI:** 10.1038/s41598-021-00232-7

**Published:** 2021-10-29

**Authors:** Yoshiyuki Miyamoto, Keigo Kubota, Yukiyo Asawa, Kazuto Hoshi, Atsuhiko Hikita

**Affiliations:** 1 Department of Sensory and Motor System Medicine, Graduate School of Medicine, The University of Tokyo, Tokyo, 113-8655 Japan; 2 Division of Dentistry and Oral Surgery, Mitsui Memorial Hospital, Tokyo, 101-8643 Japan; 3 Department of Tissue Engineering, The University of Tokyo Hospital, Tokyo, 113-8655 Japan; 4 Department of Oral-Maxillofacial Surgery, and Orthodontics, The University of Tokyo Hospital, Tokyo, 113-8655 Japan

**Keywords:** Cell biology, Immunology

## Abstract

Cartilage tissues have poor self-repairing abilities. Regenerative medicine can be applied to recover cartilage tissue damage in the oral and maxillofacial regions. However, hitherto it has not been possible to predict the maturity of the tissue construction after transplantation or to prepare mature cartilage tissues before transplantation that can meet clinical needs. Macrophages play an important role in cartilage tissue regeneration, although the exact mechanisms remain unknown. In this study, we established and verified an in vitro experimental system for the direct co-culture of cell pellets prepared from mouse auricular chondrocytes and macrophages polarized into four phenotypes (M1-like, M1, M2-like, and M2). We demonstrate that cartilage pellets co-cultured with M1-like promoted collagen type 2 and aggrecan production and induced the most significant increase in chondrogenesis. Furthermore, M1-like shifted to M2 on day 7 of co-culture, suggesting that the cartilage pellet supplied factors that changed the polarization of M1-like. Our findings suggest that cartilage regenerative medicine will be most effective if the maturation of cartilage tissues is induced in vitro by co-culture with M1-like before transplantation.

## Introduction

Cartilage disorders attribute to congenital diseases, such as cleft lip and palate, microtia, as well as trauma often cause aesthetic and functional impairments in the oral and maxillofacial regions, resulting in a decrease in patients’ quality of life. Cartilage tissues lack blood vessels and nerves and have poor self-repairing abilities once damaged. Thus, the current treatment to repair cartilage tissue damage is autologous tissue transplantation^1,2^. However, that form of treatment has several setbacks, such as the invasiveness during tissue collection, limits on the amount and type of tissue that can be collected, and deformation of transplanted cartilage tissues^3,4^.

Regenerative medicine can address these limitations. The culture technology for differentiated cells, such as epidermal cells and chondrocytes, was established by Green et al. in the 1970s^5^. Since chondrocytes can be relatively easily cultured in large quantities in vitro, cartilage regenerative medicine has become a field with relatively advanced clinical applications. However, transplantation of regenerated cartilage tissues requires a large quantity of the chondrocytes to be cultured in vitro, while the chondrocytes dedifferentiate with increased number of passages and lose their cartilage properties^6^. It is only through in vivo transplantation that immature/undifferentiated cells/tissues re-differentiate and produce matrix again to form the originally intended mature regenerated cartilage tissue^6^. However, the effects of treatment are uncertain because tissue maturity cannot be predicted even before transplantation. Transplantation of pre-matured cartilage tissue in vitro would be a more effective treatment if tissue maturation was predictable and the deformation and breakage of the construct following transplantation could be avoided.

Therefore, it is important to elucidate the maturation mechanism of regenerative tissue that is inherently present in the body.

It has been reported in recent years that macrophages play an important role in cartilage tissue regeneration^7–10^. A macrophage is a type of immune cell and is known to play different roles depending on its polarity (Supplementary information: Schema 1)^7,11–14^. M1 induced by classical activation (e.g., LPS, IFN-γ) is referred to as an inflammatory macrophage which expresses surface markers, such as CD80, CD86, CD40, and MHC-II, and promotes immune activation and inflammation for protecting against infections and pathogens by producing various inflammatory cytokines and chemokines^15–17^. Meanwhile, M2 induced by selective activation (e.g., IL-4, IL-10, IL-13) is referred to as an anti-inflammatory macrophage, and it expresses surface markers, such as CD206, CD163, and ARG-1, and promotes anti-inflammatory functions, such as immunosuppression/tolerance, immune control, and tissue regeneration by producing various anti-inflammatory cytokines and chemokines^18–20^. In osteoarthritis, a degenerative disease of joint cartilage, M1 secretes pro-inflammatory cytokines to degrade the cartilage, whereas M2 secretes pro-chondrogenic cytokines, such as TGF-β, to induce chondrogenesis^7^. It has also been reported that anti-inflammatory macrophages contribute to collagen turnover to maintain extracellular matrix homeostasis^8^. We have previously shown that M1 is dominant during the early phase of regeneration after cartilage transplantation and the polarity of macrophages gradually transit to M2 during cartilage regeneration^9,10^. This transition of macrophage polarity from M1 to M2 is also observed in the wound healing process, suggesting the existence of common mechanisms in tissue regeneration^21,22^. We have also shown that controlling the action of M1 is important for cartilage tissue regeneration, indicating the suppressive role of M1 in tissue regeneration^9^. Conversely, previous findings have shown that topical administration of M2 did not promote tissue regeneration in a skin wound healing model^23^. Although these studies show that macrophages play important roles in cartilage tissue regeneration, the modes of action of M1 and M2 remain to be fully elucidated and the types of macrophages associated with various phases of chondrogenesis are yet to be identified.

In this study, we established an experimental system for the direct co-culture of cartilage pellets made from mouse auricular chondrocytes and macrophages that were induced to differentiate into four polarities (M1-like, M1, M2-like, M2). The objective of this study was to elucidate the mutual relationship between macrophages and cartilage tissues via histological evaluations and genetic analysis for future establishment of an in vitro chondrogenesis method.

## Results

### Establishment of the co-culture system for cartilage pellets and macrophages

#### Cartilage pellets

**Figure 1:**
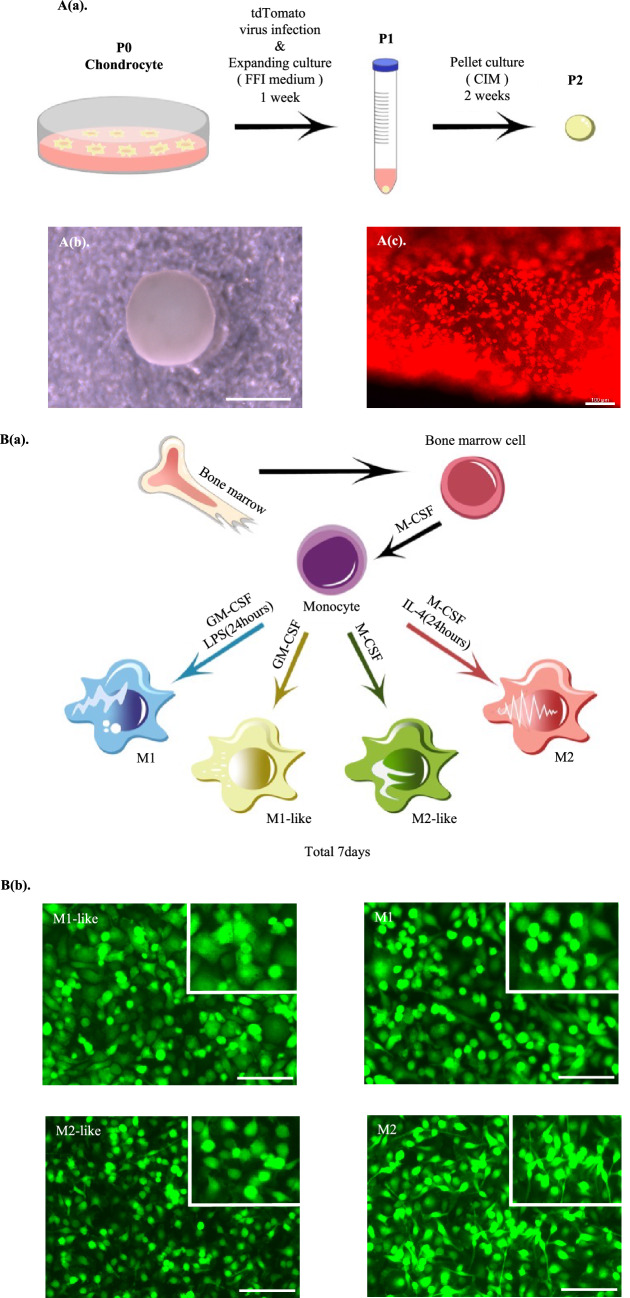

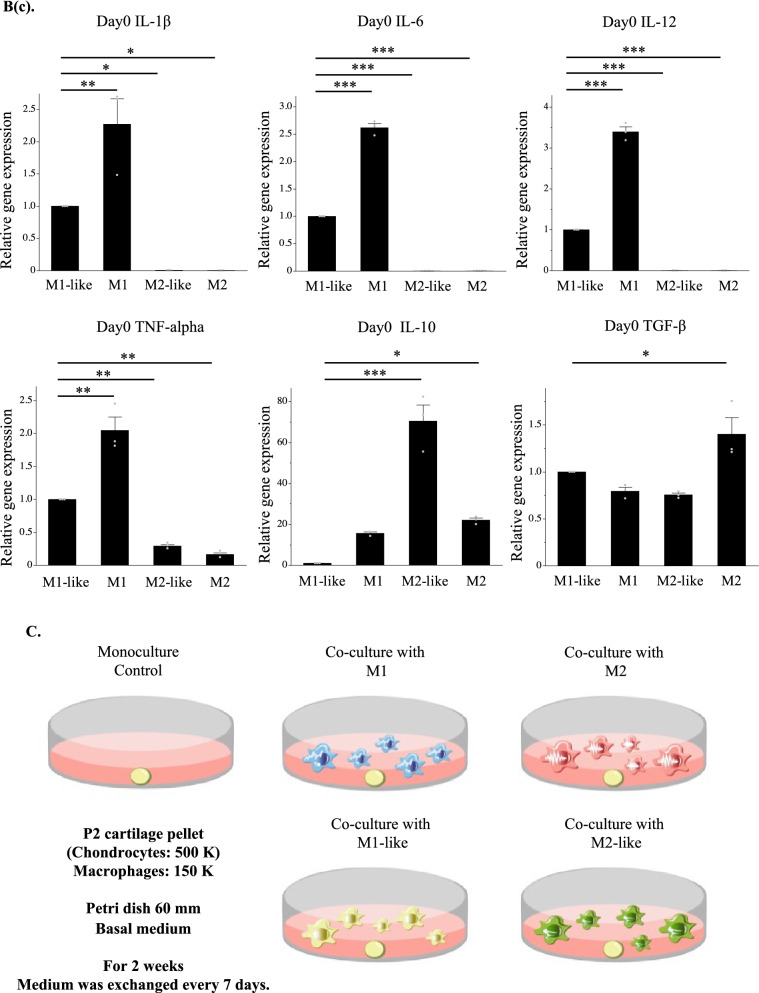
Establishment of co-culture system for cartilage pellets and macrophages. (**A**(**a**)) Cartilage pellet culturing method. (**A**(**b**)) Whole image of a cartilage pellet. Scale bar: 1 mm. (**A**(**c**)) Fluorescence microscopy findings of cartilage pellet. Magnification: 100 × , Scale bar: 100 μm. (**B**(**a**)) Macrophage differentiation culturing method. (**B**(**b**)) Fluorescence microscopy findings of macrophages. Magnification: 100 × . Scale bars: 100 μm. (**B**(**c**)) Cytokine gene expression in macrophages after induction of differentiation (day 0). *P < 0.05, **P < 0.01, ***P < 0.0001. The data are shown as the mean ± SEM (n = 3). (**C**) Co-culture system of cartilage pellets and macrophages.

The P2 cartilage pellets for which pellet culture was conducted for 2 weeks in a 15-mL conical tube (Fig. 1A(a)) showed a white color visible to the naked eye and an elastic spherical shape with a diameter of approximately 1.5 mm (Fig. 1A(b)). Fluorescence microscopy confirmed that red fluorescence of the transfected was emitted with the tdTomato gene transfer (Fig. 1A(c)).

#### Macrophages

Various factors were used to induce the differentiation of macrophages into four polarities to determine which macrophage type contributes to chondrogenesis (Fig. 1B(a)). The morphology of macrophages after the induction of differentiation was a mixture of round and spindle-shaped cells; however, many round cells were found to be M1-like, M1, and M2-like, whereas spindle-shaped cells were M2 (Fig. 1B(b)).

Real-time PCR (henceforth, RT-PCR) was used to evaluate whether there were differences in cytokine gene expression between the four macrophage types after differentiation. Results showed that M1 was highest among all inflammatory cytokines, followed by M1-like. For anti-inflammatory cytokines that were produced by macrophages and involved in cartilage formation, M2-like and M2 were significantly higher than M1-like in IL-10, and M2 was significantly higher than M1-like in TGF-β (Fig. 1B(c)).

#### Co-culture system of cartilage pellets and macrophages

Direct co-culture of the cartilage pellets and the macrophages induced to differentiate into four polarities (M1-like, M1, M2-like, and M2) was conducted (Fig. 1C).

### Cartilage pellets co-cultured with M1-like increased the production of collagen type 2 and aggrecan and exhibited the highest extent of chondrogenesis

**Figure 2:**
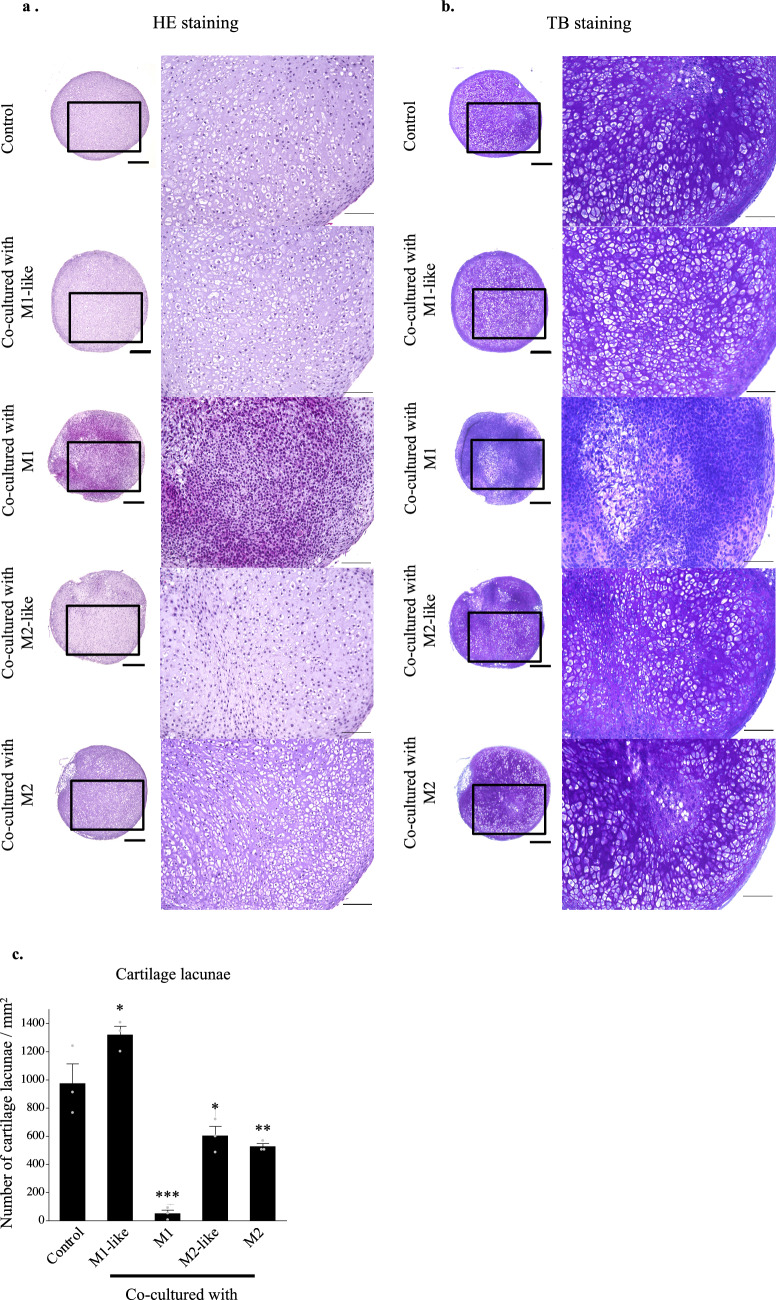

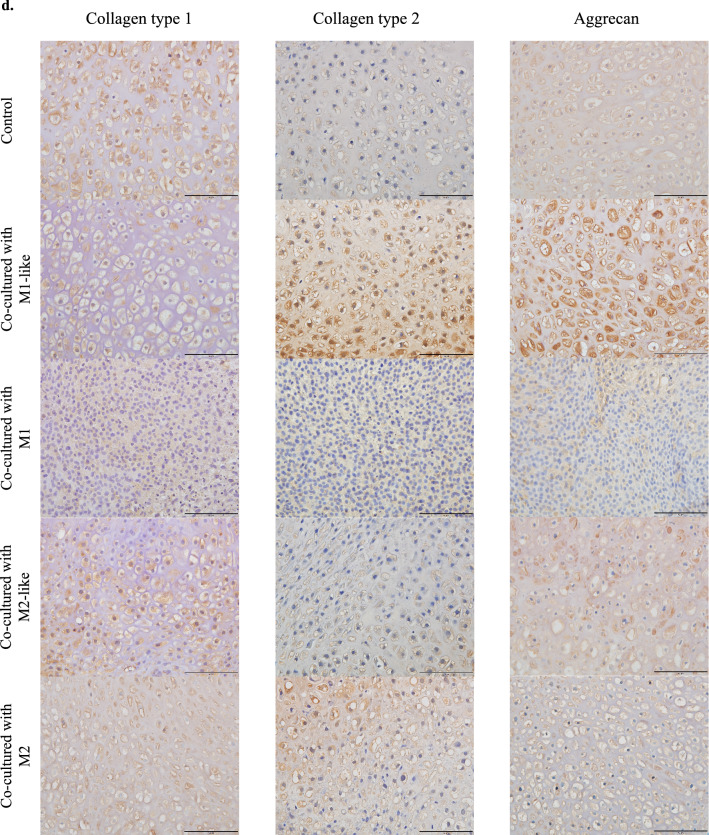

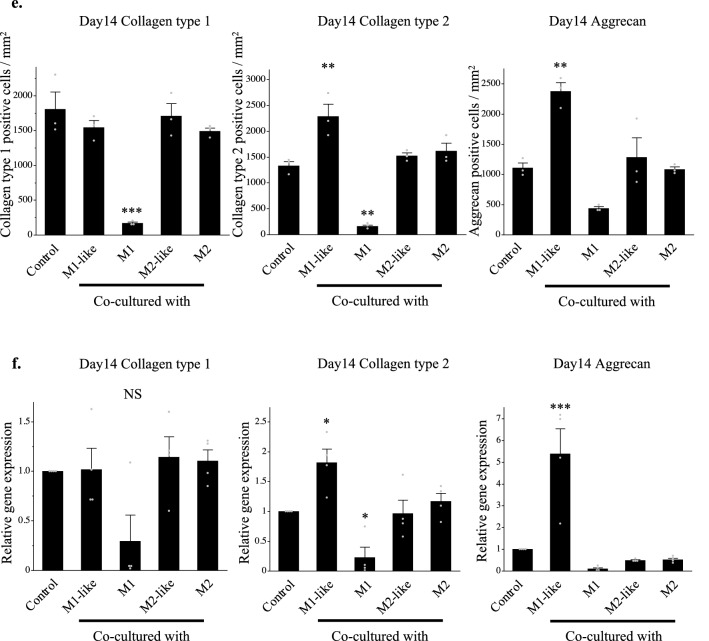
Histological findings and RT-PCR gene expression results for the cartilage pellets on day 14 of co-culture. (**a**) HE staining for cartilage pellets on day 14 of co-culture. Left: Magnification: 40 × , Scale bars: 200 μm. Right: Magnification: 200 × , Scale bars: 100 μm. (**b**) TB staining for cartilage pellets on day 14 of co-culture. Left: Magnification: 40 × , Scale bars: 200 μm. Right: Magnification: 200 × , Scale bars: 100 μm. (**c**) Evaluation of the number of cartilage lacunae using TB staining. *P < 0.05, **P < 0.01, ***P < 0.0001. The data are shown as the mean ± SEM (n = 3). (**d**) Evaluation of the matrix proteins using immunohistochemical staining. Left: Collagen type 1, Middle: Collagen type 2, Right: Aggrecan. Magnification: 400 × , Scale bars: 100 μm. (**e**) Quantitative evaluation based on the DAB-positive cell count in immunohistochemical staining (on day 14 of co-culture). Left: Collagen type 1, Middle: Collagen type 2, Right: Aggrecan. **P < 0.01, ***P < 0.0001. NS: Not significant. The data are presented as the mean ± SEM (n = 3). (**f**) Gene expression analysis using RT-PCR (on day 14 of co-culture). Left: Collagen type 1, Middle: Collagen type 2, Right: Aggrecan. The data are presented as the mean ± SEM (n = 4).

Matured auricular cartilage contained large quantities of collagen type 2 and aggrecan in the cartilage matrix, an abundance of elastic fibers, and many cartilage lacunae^24^ (Supplementary information: Schema 2). Histological evaluation of cartilage pellets on day 14 of co-culture was conducted with reference to these histological images. Cartilage lacunae were observed in the cartilage pellets of the control and those co-cultured with M1-like/M2-like/M2 in hematoxylin and eosin (HE) staining. However, there were almost no cartilage lacunae in the cartilage pellets co-cultured with M1, with densely accumulated chondrocytes (Fig. 2(a)). Predominantly metachromasia-positive matrices with relatively small cartilage lacunae were observed in certain parts of the control cartilage pellets and those co-cultured with M2-like/M2 using toluidine blue (TB) staining, representing a relatively immature tissue with active cartilage matrix production. Meanwhile, cartilage pellets co-cultured with M1-like exhibited a uniformly metachromasia-positive matrix region with relatively large cartilage lacunae, indicating a more matured tissue than other groups. Conversely, cartilage pellets co-cultured with M1 had almost no cartilage lacunae or metachromasia-positive matrix region (Fig. 2(b)). Quantitative evaluation based on the number of cartilage lacunae using TB staining revealed that cartilage pellets co-cultured with M1-like had significantly higher number of cartilage lacunae than the control, whereas significantly lower number of cartilage lacunae was observed in the co-culture of M1/M2-like/M2 and cartilage pellets. This result showed that the cartilage pellets co-cultured with M1-like had the most mature cartilage (Fig. 2(c)). Evaluation of the cartilage matrix with immunohistochemical staining of collagen type 1, collagen type 2, and aggrecan showed several DAB-positive cells of collagen type 2 and aggrecan in the cartilage pellets co-cultured with M1-like (Fig. 2(d)).

Quantitative evaluation based on the DAB-positive cell count in immunohistochemical staining revealed that cartilage pellets co-cultured with M1 had significantly lower number of cells positive for collagen type 1 and collagen type 2 than the control pellets. Meanwhile, the numbers of cells positive for collagen type 2 and aggrecan were significantly higher in the cartilage pellets co-cultured with M1-like than in the control pellets (Fig. 2(e)). Evaluation of gene expression of the cartilage pellets on day 14 of co-culture using RT-PCR showed no significant difference among all groups for collagen type 1; gene expression of collagen type 2 and aggrecan were significantly higher in the cartilage pellets co-cultured with M1-like than those in the control (Fig. 2(f)).

Thus, histological and genetic evaluations showed that the co-culture of cartilage pellets with M1-like induced the highest extent of chondrogenesis.

### Co-culture with cartilage pellets changed macrophage polarization over time

**Figure 3:**
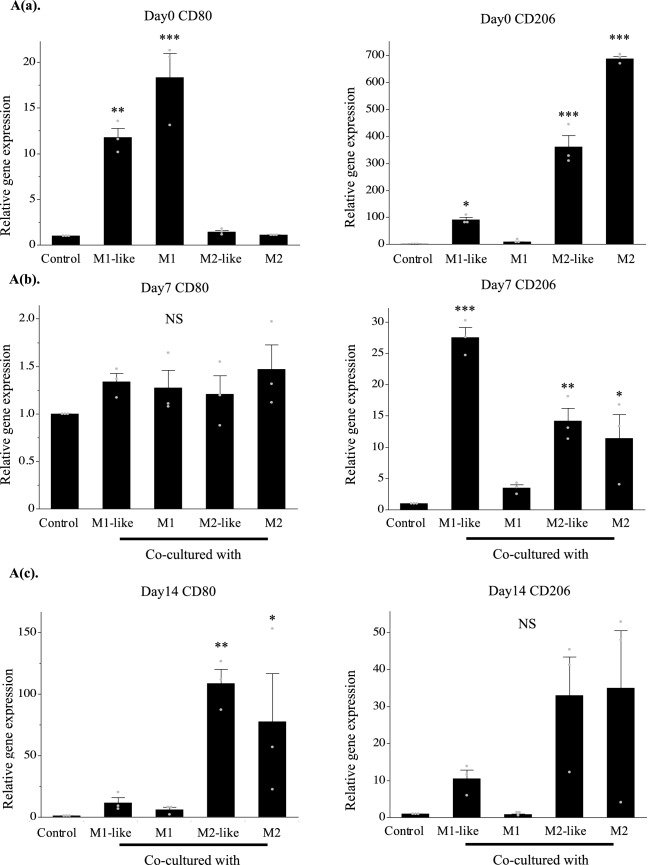

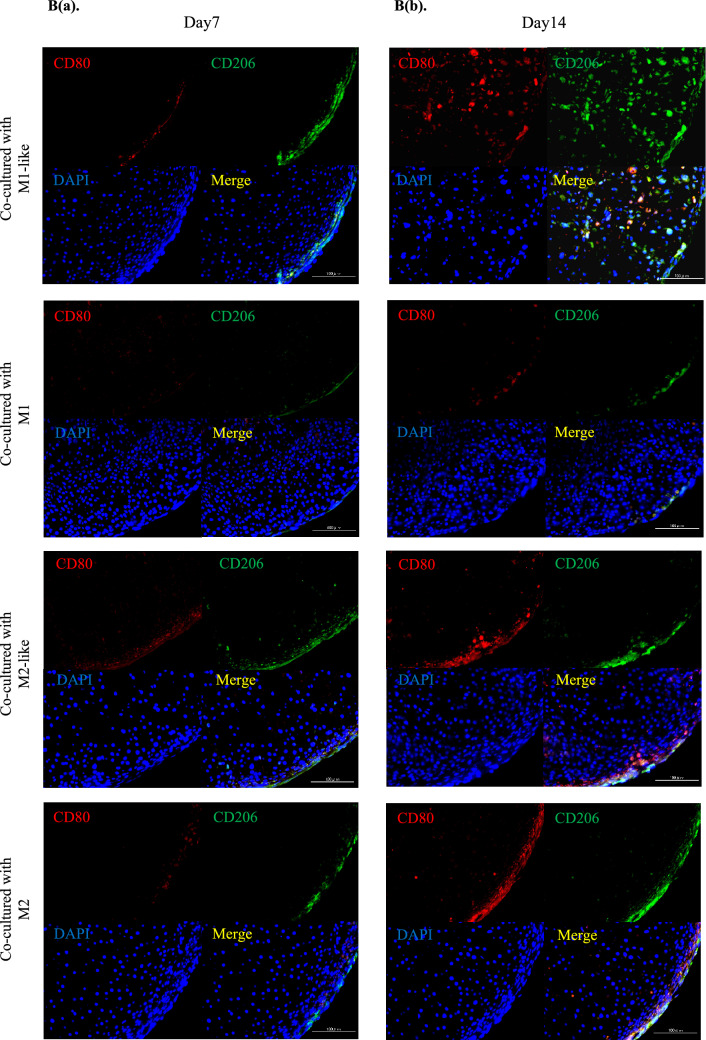

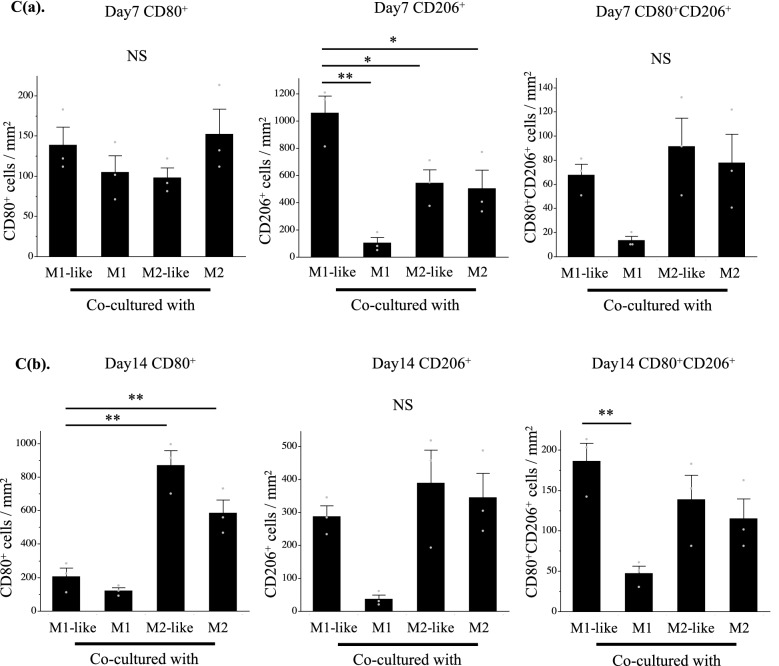
Changes in polarity of macrophages over time in co-culture. (**A**(**a**)) Gene expression in macrophages after induction of differentiation (day 0). (**A**(**b**)) Gene expression in co-culture of macrophages and cartilage pellets on day 7. (**A**(**c**)) Gene expression in co-culture of macrophages and cartilage pellets on day 14. *P < 0.05, **P < 0.01, ***P < 0.0001. NS: Not significant. The data are shown as mean ± SEM (n = 3). Left: CD80, Right: CD206. (**B**(**a**)) Double immunofluorescence analysis results for the cartilage pellets on day 7 of co-culture. (**B**(**b**)) Double immunofluorescence analysis results for the cartilage pellets on day 14 of co-culture. Double immunofluorescence staining performed for CD80 (Red: Alexa fluor 647), CD206 (Green: Alexa Fluor 488), and nuclei (blue: DAPI). Magnification: 400 × , Scale bars: 100 μm. (**C**(**a**)) Quantitative evaluation based on the CD80^+^, CD206^+^, and CD80^+^CD206^+^ cell count in double immunofluorescence staining (on day 7 of co-culture). Left: CD80^+^cells, Middle: CD206^+^cells, Right: CD80^+^CD206^+^cells. *P < 0.05, **P < 0.01. NS: Not significant. The data are presented as the mean ± SEM (n = 3). (**C**(**b**)) Quantitative evaluation based on the CD80^+^, CD206^+^, and CD80^+^CD206^+^cell count in double immunofluorescence staining (on day 14 of co-culture). Left: CD80^+^cells, Middle: CD206^+^cells, Right: CD80^+^CD206^+^cells. **P < 0.01. NS: Not significant. The data are presented as the mean ± SEM (n = 3).

Although macrophages induced into each polarity maintained their characteristics until day 7, these characteristics were not maintained on day 14 when cultured without inducing factors (Supplementary Figure 1(SI. 1)). Genetic evaluations were conducted using RT-PCR to investigate the changes in the polarization of the macrophages over time in the co-culture. After induction of differentiation (day 0), expression of the M1 surface marker CD80 was significantly higher in the culture of M1-like/M1 than in the control, while expression of the M2 surface marker CD206 was significantly higher in the culture of M1-like/M2-like/M2 than in the control (Fig. 3A(a)). CD206 expression was highest in the co-culture of M1-like and cartilage pellets on day 7 of co-culture, and CD80 expression exhibited no significant differences among all groups (Fig. 3A(b)). No significant differences were observed among all groups for CD206 on day 14 of co-culture, whereas significantly higher expression of CD80 was observed in the co-culture of M2-like/M2 and cartilage pellets (Fig. 3A(c)). Furthermore, double immunofluorescence analysis was conducted using cartilage pellets on days 7 and 14 of co-culture to evaluate the localization and distribution of macrophages. Macrophages, mostly CD206^+^, were distributed on the surface layer of cartilage pellets co-cultured with M1-like/M2-like/M2 on day 7 of co-culture (Fig. 3B(a)). On day 14 of co-culture, CD80^+^, CD206^+^ and CD80^+^CD206^+^ cells (double positive cells) were observed in all groups. However, a lower macrophage count was observed in cartilage pellets co-cultured with M1 than other groups. Their distributions were restricted on the surface layer in the cartilage pellets co-cultured with M1, M2-like and M2. Macrophages were distributed not only on the surface but also in the interior of the cartilage pellets co-cultured with M1-like (Fig. 3B(b)). We conducted quantitative analysis of macrophage polarization using double fluorescence immunostaining images. Results showed that on day 7 of co-culture, the number of CD206^+^ cells was significantly higher in cartilage pellets that were co-cultured with M1-like; no significant differences were observed among all groups for CD80^+^ and double positive cells (Fig. 3C(a)). On day 14 of co-culture, no significant differences were observed among all groups for CD206^+^ cells; the number of CD80^+^ cells in the cartilage pellets that were co-cultured with M2-like and M2 was significantly higher than that in the cartilage pellets that were co-cultured with M1-like; the number of double positive cells was significantly lower in cartilage pellets that were co-cultured with M1 than that in cartilage pellets that were co-cultured with M1-like (Fig. 3C(b)). Two-photon microscopy was used to observe the cartilage pellets on day 14 of co-culture to determine the distribution of macrophages and their contact to the cartilage pellets as well as the collagen fibers. Collagen fibers could be confirmed by SHG in all cartilage pellets. Numerous macrophages were distributed in the cartilage pellets co-cultured with M1-like/M2-like/M2, while relatively few macrophages were observed in the cartilage pellets co-cultured with M1 (Supplementary Fig. 2a (SI. 2a)). The XY, YZ, and XZ axis images showed that the macrophages were attached on the surfaces of cartilage pellets co-cultured with M1/M2-like/M2, whereas the macrophages were distributed inside the cartilage pellets co-cultured with M1-like (Supplementary Fig. 2b (SI. 2b)).

These results indicated that M1-like shifted to M2 at a relatively early stage of co-culture with the cartilage pellets. Furthermore, macrophage originated from M1-like reached a deeper layer of the cartilage pellet than the other groups on day 14 of co-culture. Meanwhile, M1 could not maintain its characteristics, and M2-like/M2 were able to maintain their characteristics until day 7 of co-culture, while these shifted to M1 on day 14 of co-culture.

### Expressions of the anti-inflammatory cytokine were significantly higher and inflammatory cytokines were suppressed in the cartilage pellets co-cultured with M1-like

Gene expression of the inflammatory cytokine IL-1β and anti-inflammatory cytokines IL-1RA and IL-10 were evaluated using RT-PCR on days 7 and 14 of co-culture.

**Figure 4:**
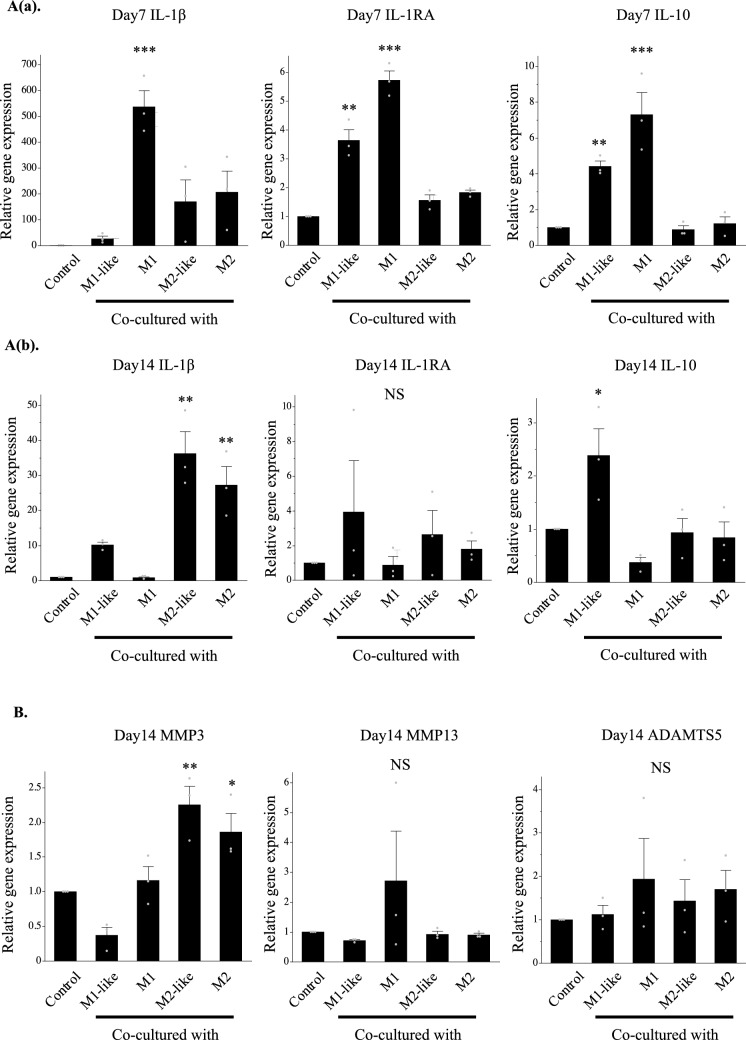

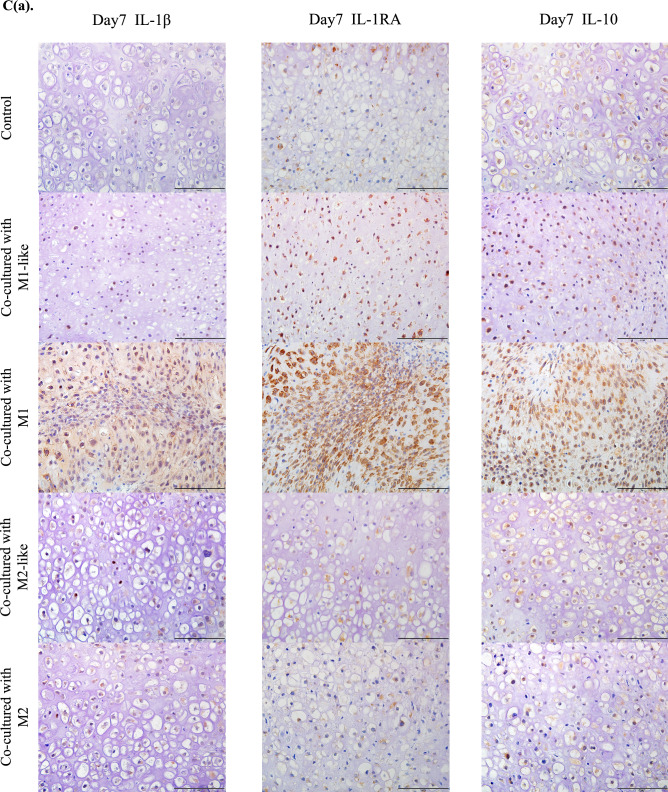

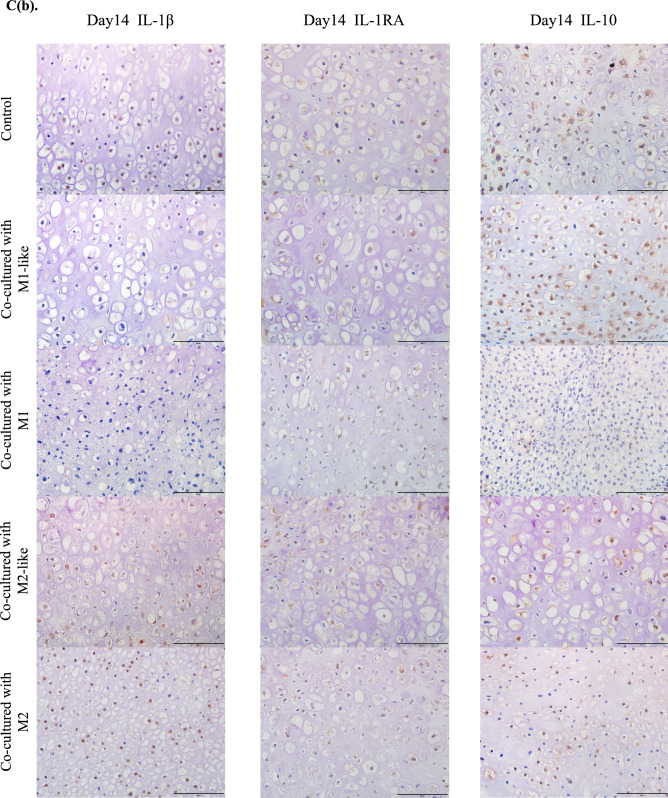

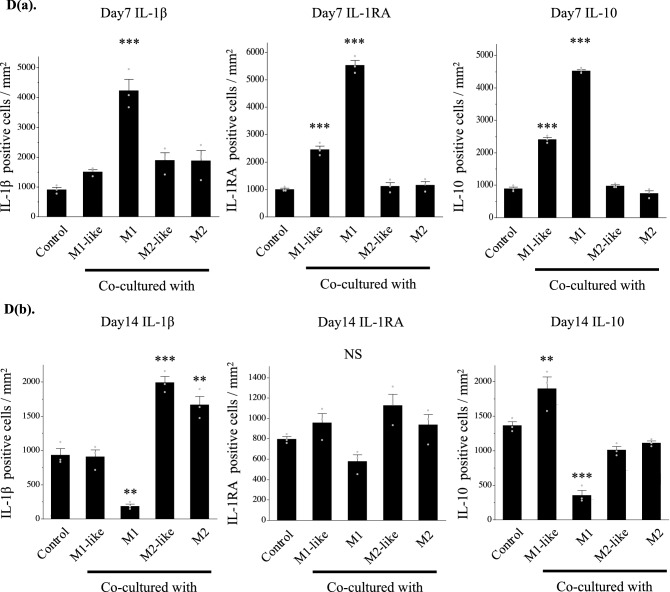
Expression analysis of cytokines in the co-culture using RT-PCR and immunohistochemistry. (**A**(**a**)) Gene expression analysis of cytokines on day 7 of co-culture using RT-PCR. (**A**(**b**)) Gene expression analysis of cytokines on day 14 of co-culture using RT-PCR. Left: IL-1β, Middle: IL-1RA, Right: IL-10. **P < 0.01, ***P < 0.0001. NS: Not significant. The data are shown as the mean ± SEM (n = 3). (**B)** Gene expression analysis of proteolytic enzymes on day 14 of co-culture using RT-PCR. Left: MMP3, Middle: MMP13, Right: ADAMTS5. *P < 0.05, **P < 0.01. NS: Not significant. The data are shown as the mean ± SEM (n = 3). (**C**(**a**)) Evaluation of cytokine production using immunohistochemical staining (on day 7 of co-culture). Left: IL-1β, Middle: IL-1RA, Right: IL-10. Magnification: 400 × , Scale bars: 100 μm. (**C**(**b**)) Evaluation of cytokine production using immunohistochemical staining (on day 14 of co-culture). Left: IL-1β, Middle: IL-1RA, Right: IL-10. Magnification: 400 × , Scale bars: 100 μm. (**D**(**a**)) Quantitative evaluation of cytokine expression based on the DAB-positive cell count in immunohistochemical staining (on day 7 of co-culture). Left: IL-1β, Middle: IL-1RA, Right: IL-10. ***P < 0.0001. The data are presented as the mean ± SEM (n = 3). (**D**(**b**)) Quantitative evaluation of cytokine expression based on the DAB-positive cell count in immunohistochemical staining (on day 14 of co-culture). Left: IL-1β, Middle: IL-1RA, Right: IL-10. **P < 0.01, ***P < 0.0001. NS: Not significant. The data are presented as the mean ± SEM (n = 3).

Gene expression of IL-1β was significantly higher in the cartilage pellets co-cultured with M1 on day 7 of co-culture, and gene expressions of IL-1RA and IL-10 were significantly higher in the cartilage co-cultured with M1-like/M1(Fig. 4A(a)). Gene expression of IL-1β was significantly higher in the cartilage pellets co-cultured with M2-like/M2 on day 14 of co-culture. Although gene expression of IL-1RA was not significantly higher in any of the groups, gene expression of IL-10 was significantly higher in the cartilage pellets co-cultured with M1-like (Fig. 4A(b)).

Evaluation of gene expression of the proteolytic enzymes involved in cartilage catabolism on day 14 of co-culture revealed significantly higher MMP3 expression in the cartilage pellets co-cultured with M2-like/M2 than in the control, while it was low in cartilage pellets co-cultured with M1-like, although not significantly. No significant differences were found in MMP13 and ADAMTS5 for all groups (Fig. 4B). Changes in cytokine protein expression were analyzed through immunohistochemical staining of cartilage pellets on days 7 and 14 of co-culture. Results showed that on day 7 of co-culture, many DAB-positive cells were observed in the cartilage pellets that were co-cultured with M1 in IL-1β, and in the cartilage pellets that were co-cultured with M1-like/M1 in IL-1RA and IL-10 (Fig. 4C(a)). On day 14 of co-culture, many DAB-positive cells were found in the cartilage pellets that were co-cultured with M1-like in IL-10, and in the cartilage pellets that were co-cultured with M2-like/M2 in IL-1β (Fig. 4C(b)). Quantitative analysis of the number of DAB-positive cells was conducted using images of immunohistochemical staining. Similar to the genetic analysis, on day 7 of co-culture, IL-1β expression was significantly higher in the cartilage pellets that were co-cultured with M1 compared to that in the control, and IL-1RA and IL-10 expressions were significantly higher in the cartilage pellets that were co-cultured with M1-like/M1 compared to that in the control (Fig. 4D(a)). Furthermore, on day 14 of co-culture, no significant differences were observed among all groups in IL-1RA, and IL-10 expression was significantly higher in the cartilage pellets that were co-cultured with M1-like and significantly lower in the cartilage pellets that were co-cultured with M1 compared to that in the control. IL-1β expression was significantly higher in the cartilage pellets that were co-cultured with M2-like and M2 and it was significantly lower in the cartilage pellets that were co-cultured with M1 compared to that in the control, as indicated in the genetic analysis (Fig. 4D(b)).

## Discussion

Macrophages were first reported in 1892 by Metchnikoff et al. as cells with cellular immune function and phagocytotic activity^25^. Milles et al. subsequently proposed the classification of macrophages into M1 and M2 phenotypes according to their functions^26^. Generally, M1 is an inflammatory macrophage that promotes inflammation and protects against infection and pathogens, whereas M2 is an anti-inflammatory macrophage that mitigates inflammation and promotes tissue regeneration^27^. However, it has been reported that, due to complex in vivo microenvironmental changes, macrophages do not exist in a state where they can be completely distinguishable between M1 and M2 phenotypes, instead exhibiting plasticity between the two polarities^16,27–30^. Furthermore, based on previous findings that topical administration of M2 did not improve wound healing^23^ and our previous study indicating that early suppression of M1 actions is important for chondrogenesis^9^, macrophages with different polarization will play various roles at different times during the tissue regeneration process.

With this in mind, this study established and verified a direct co-culture system in vitro to identify the macrophages contributing to chondrogenesis and the associated mechanisms in the mutual relationship between cartilage and macrophages. Collagen type 2 and aggrecan production was promoted and the highest extent of chondrogenesis was exhibited in cartilage pellets co-cultured with M1-like.

Although macrophages derived from monocytes using GM-CSF are referred to as M1-like due to their increased inflammatory cytokine expression^31,32^, they are known to have both M1 and M2 characteristics^33–35^. In this study, M1-like exhibited a lower tendency for inflammatory cytokine expression when compared to M1 (Fig. 1B(c)). RT-PCR revealed that they had high gene expressions of both CD80 and CD206 (Fig. 3A(a)).

Additionally, although macrophages cultured without differentiation-inducing factors maintained their characteristics until day 7 (Supplementary Fig. 1 (SI. 1)), M1-like shifted to M2 on day 7 when co-cultured with cartilage pellets (Fig. 3B(a)). These results suggest that factors that promote the M2 shift of macrophages were supplied from the cartilage pellets. An extremely large number of factors, such as cytokines (e.g., IL-4, IL-6, IL-10, and IL-13), exosomes derived from bone marrow mesenchymal stem cells and mesenchymal stromal cells, TGF-β, and adenosine, have been reported as factors influencing shift in macrophage polarization from M1 to M2 phenotypes^7,11,15,28,36–42^. In this study, IL-10 gene expression was significantly higher on day 7 of co-culture. This environment where IL-10 is highly may have promoted the shift from M1-like to M2.

Furthermore, migratory activity into the tissue has been reported to be higher in M2 than M1^19,20,43^. It has also been reported that IL-1RA increases macrophage migration^44^. In the present study, macrophages were not distributed inside the cartilage pellets in any groups on day 7 of co-culture. Meanwhile, macrophages were observed within the cartilage pellets co-cultured with M1-like on day 14 of co-culture (Fig. 3B). It was suggested that the shift from M1-like to M2 by co-culturing with cartilage pellets (Fig. 3) and the environment in which IL-1RA was highly expressed (Fig. 4) possibly increased the migration of macrophages to interior of the cartilage pellets. The presence of macrophages inside the pellet resulted in a state where macrophages could more easily influence the cartilage. However, further investigations are warranted to explain why macrophage invasion into the tissue was not observed in the cartilage pellets co-cultured with M2-like or M2.

IL-10 is an anti-inflammatory cytokine primarily produced by immune cells, such as helper T cells and macrophages^45^; it promotes the synthesis of collagen type 2 and aggrecan and induces chondrogenesis by suppressing MMP3 and MMP13 production^46,47^. IL-10 gene expression was significantly higher only in the cartilage pellets co-cultured with M1-like on day 14, while gene expression of MMP3 and MMP13 was suppressed. Therefore, the highly-expressed IL-10 may have also acted on chondrocytes, promoted anabolic actions, and suppressed catabolism, thereby promoting chondrogenesis (Fig. 4).

Meanwhile, RT-PCR and double immunofluorescence analysis conducted on day 14 revealed that cartilage pellets co-cultured with M2-like/M2 exhibited increased M1 indicated by CD80 gene expression (Fig. 3). Considering the significantly higher gene expression of IL-1β and MMP3 (Fig. 4), an inflammatory reaction might have been generated thereby enhancing cartilage catabolism.

Imaging using two-photon microscopy can be used to observe living samples and detect collagen fibers without staining using SHG^48–50^. In this study, collagen fibers and macrophage distributions in cartilage pellets undergoing co-culture could also be observed using a two-photon microscopy (Supplementary Fig. 2 (SI. 2)). However, identifying and distinguishing the collagen type from the SHG obtained using two-photon microscopy in a simple manner are challenging due to its inherent characteristics. To overcome this limitation, in future studies we will evaluate the characteristics and properties of the collagen in SHG by analyzing SHG images using neural networks.

In this study, we conducted the direct co-culture of cartilage pellets and macrophages in vitro to exclude factors by other cells and elucidate the actions of macrophages on chondrogenesis. However, cells, such as vascular endothelial cells and mesenchymal stem cells, have also been reported to contribute to chondrogenesis^51,52^. Further investigations are required in order to elucidate the mutual relationships between M1-like and other cells in cartilage tissue regeneration, and the actions of M1-like in vivo. One of the limitations of this study is that, although changes in the expression of various molecules were detected in the gene expression analysis, the cell source of each molecule was unknown since chondrocytes and macrophages from same species (mouse) were simultaneously analyzed. IL-10 might have promoted the anabolic actions of chondrocytes at the same time as the macrophage shift to M2 phenotype. However, we have not fully elucidated from which cells this cytokine was secreted. Analyses of gene expression for each cell are needed in the future to further elucidate the mutual relationship between chondrocytes and macrophages.

The results obtained in this study indicated the usefulness of M1-like for in vitro maturation of regenerative cartilage. If the transplantation of regenerative cartilage matured in advance in vitro through the application of M1-like becomes possible, the deformation and breakage of transplants after transplantation can be avoided, and cartilage regenerative medicine will be further developed as an effective treatment strategy.

## Material and methods

Supplementary experimental procedure and all materials are described in Supplementary information.

### Ethical statement

This study was carried out in compliance with the ARRIVE guidelines, and all methods were carried out in accordance with relevant guidelines and regulations.

### Isolation of mouse auricular chondrocytes

All animal experiments were approved by the animal experiment committee of the University of Tokyo Graduate School of Medicine (approval number: Medical-P15-019, Medical-P19-114), and considerations were given to animal welfare based on laws and regulations, including the Act on Welfare and Management of Animals of Japan. Cartilage from the auricle and ear canal of six-week-old male C57BL6/J mice euthanized by cervical dislocation was collected, and the perichondrium was peeled off. Cartilage tissues were shaken in 0.15% collagenase solution (collagenase in Dulbecco’s Modified Eagle Medium: Nutrient Mixture F-12 medium (DMEM/F12)) in a constant temperature bath at 37 °C for 8 h. The obtained suspension was filtered through a 100-μm pore diameter cell strainer to remove the residue, followed by centrifugation at 500 × *g* for 5 min to isolate chondrocytes. The isolated chondrocytes were seeded in a 10-cm diameter collagen type 1 coating dish at a density of 50 × 10^4^ cells/dish. Cells were then cultivated in DMEM/F12 with 5% fetal bovine serum, 5 μg/mL insulin, 1% penicillin/streptomycin, and 100 ng/mL FGF2 (FBS-FGF2-insulin medium: henceforth, FFI medium) at 37 °C and 5% CO_2_. After the culture expanded in the FFI medium for 7 days, cells were collected using 0.05% Trypsin–EDTA solution, and Cellbanker was used to cryopreserve the cells at − 80 °C (Passage 0; henceforth, P0).

### Cartilage pellet culturing method

The cryopreserved chondrocytes (P0) were thawed, a total of 50 × 10^4^ cells were seeded on a 10-cm diameter collagen type 1 coating dish, and the FFI medium was used for initial cultures at 37 °C and 5% CO_2_. The next day, 5 μL of 8 mg/mL polybrene was added to 10 mL of the tdTomato virus-containing culture supernatant and filtered through a 45-μm pore cell strainer; chondrocytes were infected using a virus-containing culture solution diluted twofold using the FFI medium (Passage 1; henceforth P1). The virus-containing culture solution was entirely aspirated after 24 h, the medium was replaced with the FFI medium, and the culture was maintained at 37 °C and 5% CO_2_ until it reached 80% confluency (7 days). The cells were collected using 0.05% Trypsin–EDTA solution and washed twice in DMEM-high glucose with 1% penicillin/streptomycin. As reported by Uzielienė et al.^53–55^, cells were centrifuged at 600 × *g* for 5 min to ensure that 50 × 10^4^ cells/500 μL would be present in a single 15-mL conical tube. The resulting pellets (Passage 2; henceforth P2) were cultured for 2 weeks at 37 °C and 5% CO_2_ using a chondrocyte induction medium (CIM): DMEM-high glucose supplemented with 1% sodium pyruvate, 1% penicillin/streptomycin, 1% insulin-transferrin-selenium-ethanolamine, 0.17 mM ascorbic acid phosphate, 0.35 mM L-prolin, 1 × 10^–7^ M dexamethasone, and 10 ng/mL TGF-β3. The medium was exchanged once in 2–3 days. The cartilage pellets after culturing were observed and photographed using Leica S9D, Leica DMi8, and LAS X.

### Macrophage isolation method and differentiation induction method

Mouse bone marrow cells were collected from the femur, tibia, and humerus of six-week-old male GFP mice euthanized by cervical dislocation using the flash method^56,57^. This cell suspension was then hemolyzed using filtered RBC lysis buffer and washed with 1 × PBS and filtered through a 70-μm pore cell strainer. Cells were cultured for 24 h to increase monocyte count using a culture solution of DMEM-high glucose supplemented with 10% FBS, 1% penicillin/streptomycin, and 20 ng/mL M-CSF was added to a 20-cm dish. The next day, floating monocytes were collected and filtered using a 45-μm pore cell strainer to prepare the cell suspension.

To induce the differentiation of the monocytes into four types of macrophages, we referred to Buchacher et al.^14^ and used a 15-cm petri dish to seed 500 × 10^4^ cells. A DMEM-high glucose supplemented with 10% FBS and 1% penicillin/streptomycin (henceforth, basal medium) was then prepared, to which following reagents were added for indicated periods to induce each type of macrophage phenotype (Fig. 1B(a)): [M1-like: 20 ng/mL GM-CSF for 1 week; M1: 20 ng/mL GM-CSF for 1 week and 100 ng/mL LPS for 24 h at the end of induction culture; M2-like: 50 ng/mL M-CSF for 1 week; and M2: 50 ng/mL M-CSF for 1 week and 20 ng/mL IL-4 for 24 h at the end of induction culture]. Cells were cultured for 1 week at 37 °C in 5% CO_2_, and observed and photographed with a Leica DMi8 and LAS X.

### Co-culture of cartilage pellets and macrophages

P2 cartilage pellet was prepared from 50 × 10^4^ cells, as described above, and was placed in a 60-mm petri dish. Macrophages induced to differentiate (M1-like, M1, M2-like, and M2) were seeded at a density of 15 × 10^4^ cells/dish. The dish was carefully stirred to ensure uniform distribution of macrophages, and direct co-culture was initiated with the basal medium at 37 °C in 5% CO_2_. Cartilage pellets cultured in basal medium without macrophages were used as control. The medium was replaced 1 week after co-culture, and the co-culture period was set to 2 weeks.

### Histology, immunohistochemical analysis, and double immunofluorescence analysis

#### Hematoxylin and eosin stain (HE staining) and Toluidine blue stain (TB staining)

The cartilage pellets co-cultured with macrophages and control pellets were collected on day 14 of co-culture. The samples were fixed with 4% paraformaldehyde, soaked with PBS and dehydrated, and embedded in paraffin. Tissues were sliced at 5-μm thickness using Leica RM2265, and they were subjected to HE and TB staining. Specimens were observed and photographed with the HS All-in-one Fluorescence Microscope BZ-9000 and BZ-II Analyzer. For the cartilage lacunae count, six regions were randomly selected in images observed at 200 × magnification, and the average number of cartilage lacunae per unit area was counted. Cell counting was performed by two observers.

#### Immunohistochemical analysis

Paraffin sections (5-μm thickness) were prepared as described above using samples collected on days 7 and 14 of co-culture, and immunohistochemical staining of collagen type 1, collagen type 2, aggrecan, IL-1β, IL-1RA, and IL-10 was conducted using the streptavidin–biotin method. The primary antibodies and antigen retrieval methods used are listed in Supplementary Table 1 (SI. 3). Biotinylated anti-rabbit IgG antibody was used as a secondary antibody. Color reaction detection was conducted using a Peroxidase Stain DAB Kit. Hematoxylin was used as a counterstain for all immunohistochemical staining. Specimens were observed and photographed using an OLYMPUS BX51 and cellSens ver2.3. For the DAB-positive cell count, six regions were randomly selected in images observed at 400 × magnification, and the average number of cells per unit area was counted. Cell counting was performed by two observers.

#### Double immunofluorescence analysis

For double immunofluorescence analysis, the cartilage pellets and macrophages prepared from six-week-old male C57BL/6J mice were used for co-culture to ensure that the results would not be affected by fluorescence from the GFP and tdTomato. They were co-cultured for 14 days as described above before sample collection. Paraffin sections (5-μm thickness) were de-paraffinized with xylene alcohol, incubated for 60 min using Blocking One, and reacted overnight at 4 °C with primary antibodies. The primary antibodies and antigen retrieval methods used are listed in Supplementary Table 2 (SI. 4). Afterwards, PBS-T was used to wash the samples 3 times for 5 min each. They were then incubated in a dark area for 1 h with fluorescently labeled secondary antibodies (Supplementary Table 3 (SI. 5)). Nuclear staining and encapsulation were conducted using Vectashield plus with DAPI after washing with PBS-T. Specimens were observed and photographed with an HS All-in-one Fluorescence Microscope BZ-9000 and BZ-II Analyzer. For the positive cell count, six regions were randomly selected in images observed at 400 × magnification, and the average number of cells per unit area was counted. Cell counting was performed by two observers.

### Real-time PCR (RT-PCR)

Total RNA was collected from the control (monoculture) and co-cultured cartilage pellets on days 7 and 14, as well as from macrophages after induction of differentiation (day 0) using ISOGEN in accordance with the protocol of the manufacturer. Briefly, the samples were homogenized using ISOGEN. Then, chloroform was added and the cells were centrifuged (12,000 × *g* for 15 min at 4 °C) to collect the aqueous phase which contained the RNA. The RNA was then precipitated using isopropanol and collected by centrifugation. After the addition of 70% ethanol, the samples were centrifuged again. The precipitated total RNA was then air-dried and dissolved with purified water. cDNA was prepared via reverse transcription using the PrimeScript RT reagent Kit in accordance with the manufacturer’s protocols. A 7500 Fast Real-Time PCR System was used to measure the relative gene expression levels using the ΔΔCT method. The primers that were used in the real-time PCR are shown in Supplementary Table 4 (SI. 6).

### Statistical analysis

All data were expressed as mean ± SEM. JMP Pro software version 16.0.0 was used as the statistical analysis software. One-way ANOVA was first used for multiple comparison tests, and the Dunnett’s test was used for post-hoc analysis. Statistical significance (P-value) was set at 5%, where P < 0.05 was the level at which statistical significance was defined.

## Supplementary Information

Supplementary Information.

## Data Availability

The datasets generated during the current study are available from corresponding author (A.H.) on reasonable request.
