# Publisher Correction: M1-like macrophage contributes to chondrogenesis in vitro

**DOI:** 10.1038/s41598-022-07417-8

**Published:** 2022-02-22

**Authors:** Yoshiyuki Miyamoto, Keigo Kubota, Yukiyo Asawa, Kazuto Hoshi, Atsuhiko Hikita

**Affiliations:** 1 Department of Sensory and Motor System Medicine, Graduate School of Medicine, The University of Tokyo, Tokyo, 113-8655 Japan; 2 Division of Dentistry and Oral Surgery, Mitsui Memorial Hospital, Tokyo, 101-8643 Japan; 3 Department of Tissue Engineering, The University of Tokyo Hospital, Tokyo, 113-8655 Japan; 4 Department of Oral-Maxillofacial Surgery, and Orthodontics, The University of Tokyo Hospital, Tokyo, 113-8655 Japan

Correction to: *Scientific Reports* https://doi.org/10.1038/s41598-021-00232-7, published online 29 October 2021

The original version of this Article contained errors.

In Figure 2e (left graph, “Day 14 Collagen type 1”), a grey data point located above the error bar in the “Control” bar was omitted. Additionally, in Figure 2f (middle graph, “Day 14 Collagen type 2”), a grey data point located above the error bar in the “Co-cultured with M2-like” bar was omitted.

Furthermore, in Figure 3C (a) (middle graph, “Day7 CD206^+^”) the y-axis label “0” and the grey data point located above the bar “Co-cultured with M1” were duplicated.

The original Figure 2e and 2f, and Figure 3C(a) and accompanying figure legends appears below.

**Figure 2:**
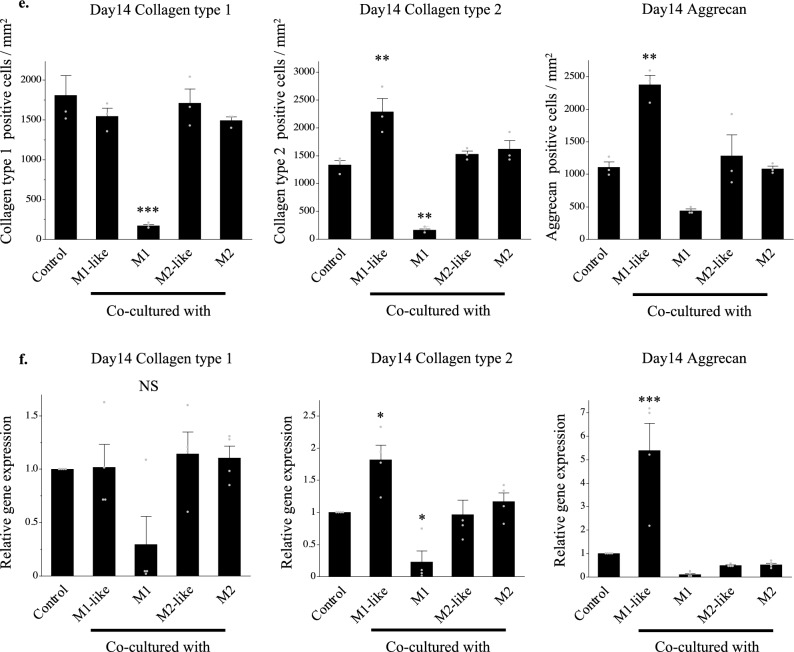
**(e)** Quantitative evaluation based on the DAB-positive cell count in immunohistochemical staining (on day 14 of co-culture). Left: Collagen type 1, Middle: Collagen type 2, Right: Aggrecan. **P < 0.01, ***P < 0.0001. NS: Not significant. The data are presented as the mean ± SEM (n = 3). **(f)** Gene expression analysis using RT-PCR (on day 14 of co-culture). Left: Collagen type 1, Middle: Collagen type 2, Right: Aggrecan. The data are presented as the mean ± SEM (n = 4).

**Figure 3:**
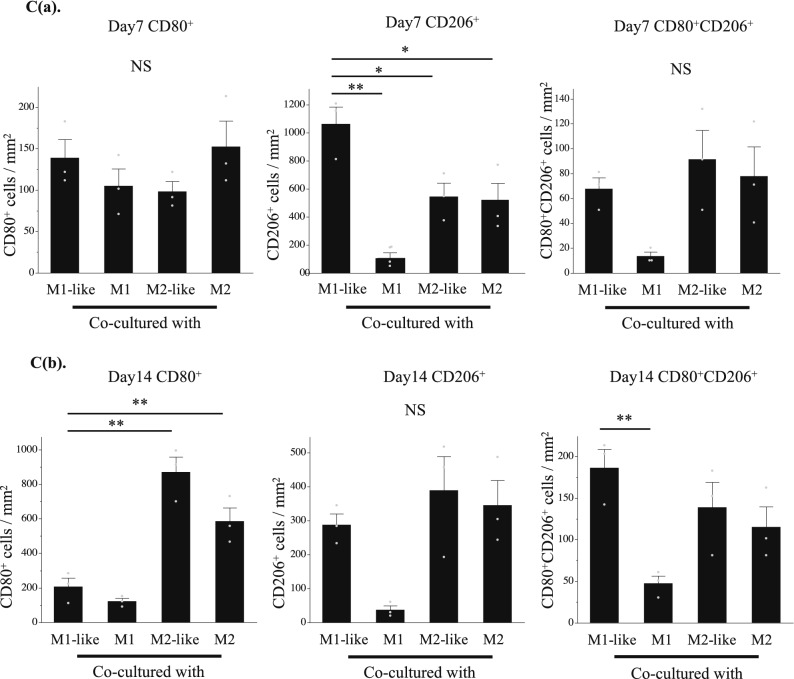
**C(a)** Quantitative evaluation based on the CD80^+^ , CD206^+^ , and CD80^+^CD206^+^ cell count in double immunofluorescence staining (on day 7 of co-culture). Left: CD80^+^ cells, Middle: CD206^+^ cells, Right: CD80^+^CD206^+ ^cells. *P < 0.05, **P < 0.01. NS: Not significant. The data are presented as the mean ± SEM (n = 3).

The original Article has been corrected.

