# Supplementary material for: M1-like macrophage contributes to chondrogenesis in vitro
Source: Sci Rep. 2021 Oct 29;11:21307. doi: 10.1038/s41598-021-00232-7 (PMC8556372; doi:10.1038/s41598-021-00232-7)
Supplement: Supplementary file 1 — Supplementary Information. [file 41598_2021_232_MOESM1_ESM.pdf]

Supplementary Information

M1 -like macrophage contributes to chondrogenesis in vitro

Yoshiyuki Miyamoto[1], Keigo Kubota[2,4], Yukiyo Asawa[3], Kazuto Hoshi[1,3,4]&Atsuhiko Hikita[3*]

[1]Department of Sensory and Motor System Medicine, Graduate school of Medicine, The University of Tokyo, Tokyo113-8655, Japan
[2]Division of Dentistry and Oral Surgery, Mitsui Memorial Hospital, Tokyo 101-8643, Japan
[3]Department of Tissue Engineering, The University of Tokyo Hospital, Tokyo 113-8655, Japan
[4]Department of Oral-Maxillofacial Surgery, and Orthodontics, The University of Tokyo Hospital, Tokyo 113-8655, Japan

*Correspondence and requests for materials should be addressed to A.H. (email: ahikita-tky@g.ecc.u-tokyo.ac.jp)

Supplemental experimental procedure

tdTomato gene transfer into mouse auricular chondrocytes

We complied with the rules for genetic recombination experiments and received approval (approval number; 50); the experiment was inspected by the genetically modified organisms committee of the University of Tokyo Graduate School of Medicine. The retroviral expression vector pMXs-Puro-tdTomato was reconstructed by inserting the gene for the red fluorescent protein (tdTomato) into the multicloning site of retroviral expression vector pMXs-Puro. This tdTomato-expressing retroviral vector was transfected by using Fugene6 Transfection Reagent into Platinum-E cells. After culturing for 48 hours, the virus-containing culture supernatant was used for the transfection of tdTomato gene into chondrocytes.

Two-photon microscopy

Cartilage pellets were collected from the dish on day 14 of co-culture. The pellets were lightly washed with the basal medium and transferred into a prepared mold made using a 2% agarose solution prepared by dissolving agarose for observation. Two-photon excited fluorescence images and SHG images were acquired using a multiphoton confocal microscopy system with an excitation laser (wavelengths: 690–1,040 nm; repetition rate: 80 MHz; pulse width: 70 fs) and a water-immersion objective lens (CFI75 Apo 25 ×W MP, numerical aperture: 1.1). The excitation wavelength was 950 nm. The emission filters used were as follows: 492 nm short-pass for SHG of collagen, 525/50 nm band-pass for GFP of macrophages, and 575/25 band-pass for tdTomato of chondrocytes. Images were taken at 1.5-μm intervals from the surface layer of the cartilage pellets. Images were acquired using NIS-Elements ver. 4.0. The brightness and contrast of some images were adjusted with the same parameters among relevant images to facilitate visibility, and this is indicated in the corresponding figure legends.

Materials

Isolation of mouse auricular chondrocytes

C57BL6/J mice (Nisseizai, Tokyo, Japan), collagenase (FUJIFILM Wako Pure Chemical Corporation, Osaka, Japan), Dulbecco's Modified Eagle Medium: Nutrient Mixture F-12 medium (DMEM/F12; Sigma-Aldrich Co., MO, USA), 100-µm pore diameter cell strainer (Corning Incorporated, New York, USA), 10-cm diameter collagen type 1 coating dish (AGC Techno Glass Co., Ltd., Tokyo, Japan), fetal bovine serum (Invitrogen, Carlsbad, CA), insulin (MP Biomedicals, Irvine, CA), 1% penicillin/streptomycin (Sigma-Aldrich Co., MO, USA), FGF-2 (henceforth, FFI medium; Kaken Pharmaceutical Co., Ltd., Tokyo, Japan), 0.05% Trypsin-EDTA solution (Sigma-Aldrich Co., MO, USA), Cellbanker (Zenoaq Resource, Fukushima, Japan).

td-Tomato gene transfer into mouse auricular chondrocytes

pMXs-Puro (Cell Biolabs, Inc., San Diego, USA), Fugene6 Transfection Reagent (Promega Corporation, Madison, USA).

Cartilage pellet culturing method

Polybrene (Hexadimethrine Bromide; NACALAI TESQUE, INC., Osaka, Japan), 45-µm pore cell strainer (*Corning Incorporated*, New York, USA), Dulbecco's Modified Eagle's Medium - high glucose (DMEM-high glucose; Sigma-Aldrich Co., MO, USA), 15-mL conical tube (*Corning Incorporated*, New York, USA), 1% sodium pyruvate (Life Technologies, Carlsbad, CA), 1% insulin-transferrin-selenium-ethanolamine (Life Technologies, Carlsbad, CA), ascorbic acid phosphate (Sigma-Aldrich Co., MO, USA), L-prolin (Sigma-Aldrich Co., MO, USA), dexamethasone (Sigma-Aldrich Co., MO, USA), TGF-β3 (Proteintech Group, Inc., Chicago, USA), Leica S9D/ Leica DMi8 and LAS X (Leica microsystems, Wetzlar, Germany).

Macrophage isolation method and differentiation induction method

GFP mice (CLEA Japan, Inc., Tokyo, Japan), RBC lysis buffer < adjusted with 9 mL of 0.16 M NH_4Cl (FUJIFILM Wako Pure Chemical Corporation, Osaka, Japan) and 1 mL of 0.17 M Tris (MP Bio Japan K.K., Tokyo, Japan)>, 1×PBS (FUJIFILM Wako Pure Chemical Corporation, Osaka, Japan), 70-µL pore cell strainer (*Corning Incorporated*, New York, USA), M-CSF (FUJIFILM Wako Pure Chemical Corporation, Osaka, Japan), 20-cm dish (*Corning Incorporated*, New York, USA), 15-cm petri dish (*Corning Incorporated*, New York, USA), GM-CSF (FUJIFILM Wako Pure Chemical Corporation, Osaka, Japan), LPS (FUJIFILM Wako Pure Chemical Corporation, Osaka, Japan), IL-4 (FUJIFILM Wako Pure Chemical Corporation, Osaka, Japan).

Co-culturing of macrophages and cartilage pellets

60-mm petri dish (*Corning Incorporated*, New York, USA).

Histology, immunohistochemical analysis, and double immunofluorescence analysis

4% paraformaldehyde (FUJIFILM Wako Pure Chemical Corporation, Osaka, Japan), Leica RM2265 (Leica microsystems, Wetzlar, Germany), HS All-in-one Fluorescence Microscope BZ-9000 and BZ-II Analyzer (Keyence Corp., Osaka, Japan), Biotinylated anti-rabbit IgG antibody (NICHIREI BIOSCIENCES INC., Tokyo, Japan), Peroxidase Stain DAB Kit (Nacalai, Osaka, Japan), Hematoxylin (Sakura Finetek Japan Co., Ltd., Tokyo, Japan), OLYMPUS B×51 and cellSens ver2.3 (Olympus Corporation, Tokyo, Japan), Blocking One (Nacalai, Osaka, Japan), PBS-T <prepared from 1×PBS and 0.1% Tween 20 (Nacalai, Osaka, Japan)>, Vectashield plus with DAPI (VECTOR LABORATORIES, USA).

Real-time PCR (RT-PCR)

ISOGEN (NIPPON GENE CO., LTD, Tokyo, Japan), PrimeScript RT reagent Kit (Takara Bio, Sigma, Japan), agarose (Agarose H14, Takara Bio Inc., Gunma, Japan), 7500 Fast Real-Time PCR System (Applied BioSystems, Carlsbad, CA, USA).

Two-photon microscopy

Agarose (Agarose H14, Takara Bio Inc., Gunma, Japan), multiphoton confocal microscopy system (A1R + MP, Nikon Corporation, Tokyo, Japan), excitation laser (Mai Tai eHP, Spectra-Physics, Tokyo, Japan), a water-immersion objective lens (CFI75 Apo 25×W MP, numerical aperture: 1.1, Nikon Corporation, Tokyo, Japan), NIS-Elements ver. 4.0 (Nikon Corporation, Tokyo, Japan).

Statistics

JMP Pro software version 16.0.0 (SAS Institute, North Carolina, USA).

Schema

Schema 1. M1 and M2 characteristics.

Schema 2. Histological characteristics of mouse auricular cartilage.

Matured chondrocytes include a large quantity of collagen type 2 and aggrecan in their cartilage matrix, an abundance of elastic fibers, and cartilage lacunae can also be found.

Schema 1.

M1

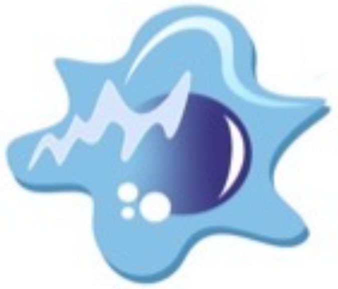

Inflammatory macrophages

Stimuli: LPS, IFN-γ, TNF-α

Released Product : TNF-α, IL-1β, IL-6, IL-12, IL-23, NO, MMP-1, MMP-3, MMP-13, ADAMTS

Surface marker: CD80, CD86, CD40

M2

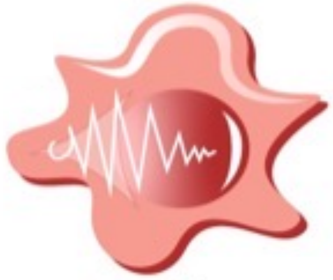

Anti-inflammatory macrophages

Stimuli: IL-4, IL-13, IL-10

Released Product : IL-10, IL-1RA, TGF-β, IGF, MMP-1, MMP-12

Surface marker: CD206, CD163, ARG-1

Schema 2.

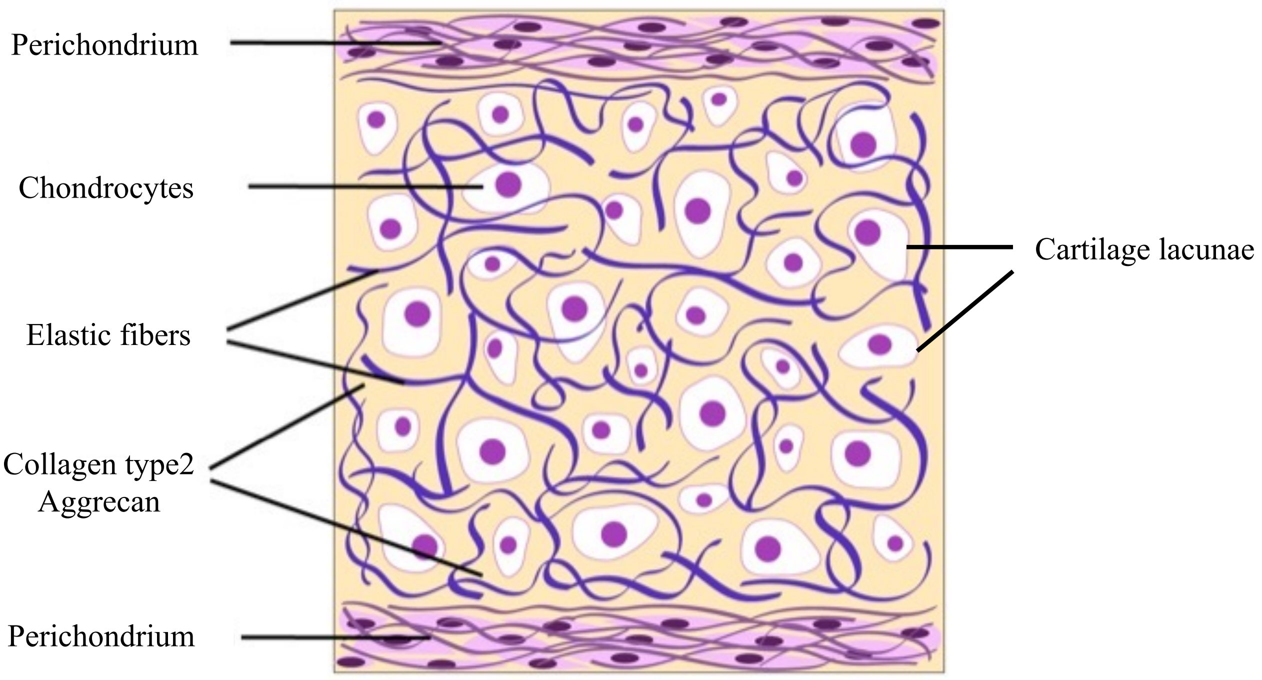

Supplementary Figure. 1. (SI. 1.)

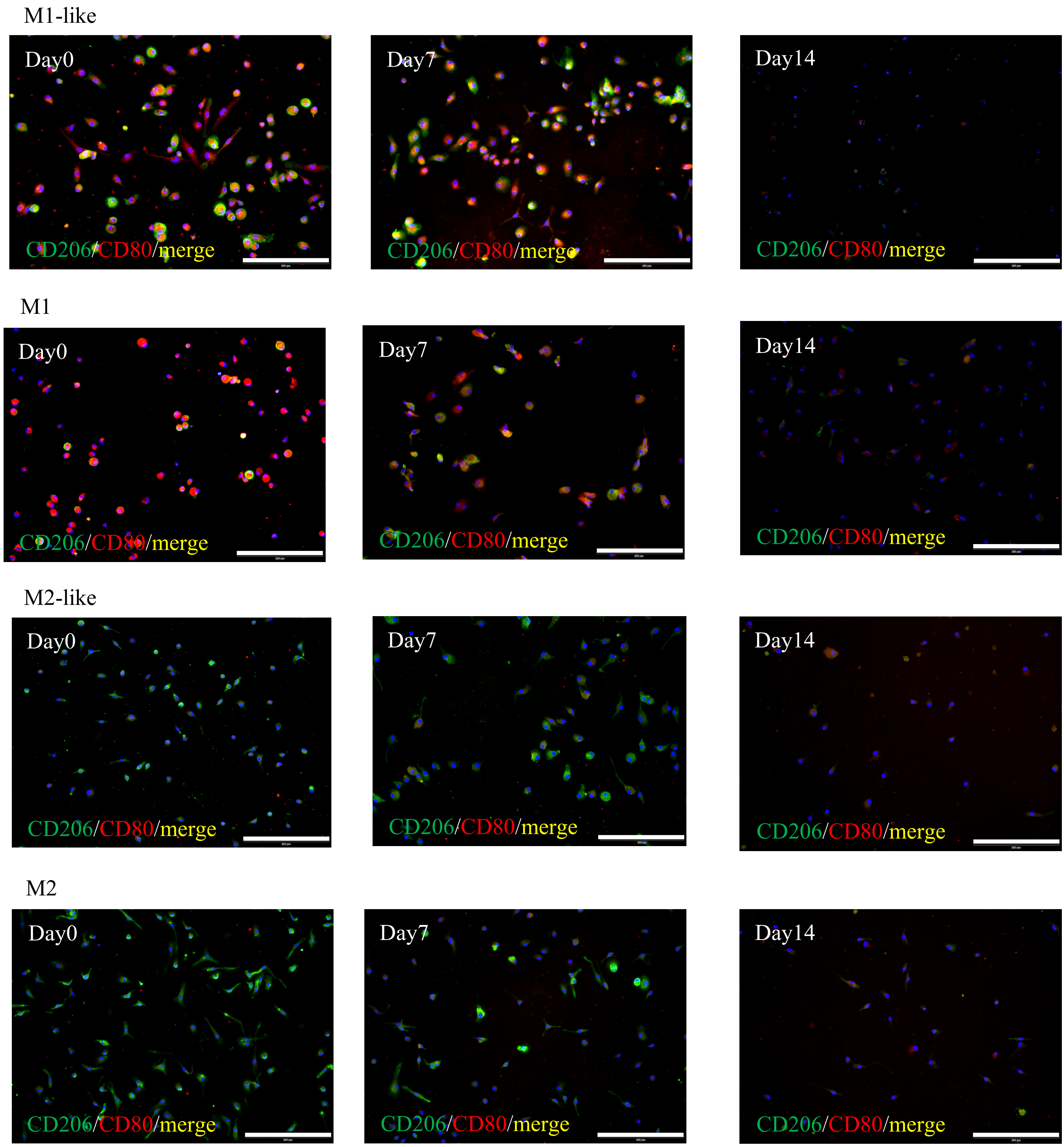

Supplementary Figure 1. (SI. 1)

Changes in polarity of macrophages over time in monoculture.

Left: day 0, Middle: day 7, Right: day 14

Double immunofluorescence staining performed with CD80 (Red: Alexa fluor 594), CD206 (Green: Alexa Fluor 488), and DAPI for staining nuclei (blue).

Three independent experiments were carried out, and a representative experiment is shown.

Magnification: 400x, Scale bars: 100 µm

Method (SI. 1.)

Double immunofluorescence analysis

Double immunofluorescence analysis was conducted using macrophages, used for monoculture, prepared from six-week-old male C57BL/6J mice, such that that the results remained unaffected by fluorescence from the GFP and tdTomato. Macrophages after induction of differentiation were seeded with a cell count of 15×10^4 on a Chamber Slide (Watson Bio Lab, Kobe, Japan), and a basal medium was used to start culturing under the conditions of 37℃ and 5% CO_2. Double immunofluorescence analysis was conducted on day 0 (8 hours after culturing), day 7, and day 14. Briefly, after aspirating the culture solution and washing with 1x PBS, cells were fixed with 4% paraformaldehyde for 10 minutes and washed again with 1x PBS. Tissue penetration treatment was performed with PBS-T for five minutes. After incubating for 60 minutes using Blocking One (Nacalai, Osaka, Japan), a primary antibody was used to react at room temperature for one hour. The primary antibodies used are shown in Supplement Table 2 (SI.4). This was washed with 1x PBS and reacted in a dark location for one hour with a fluorescently-labeled secondary antibodies (Supplement Table 5 (SI. 7)). Nuclear staining and encapsulation were conducted using Vectashield plus with DAPI after washing with PBS-T. Specimens were observed and photographed with a HS All-in-one Fluorescence Microscope BZ-9000 and BZ-II Analyzer (Keyence Corp., Osaka, Japan).

Supplementary Figure. 2. (SI. 2.)

SI. 2a.

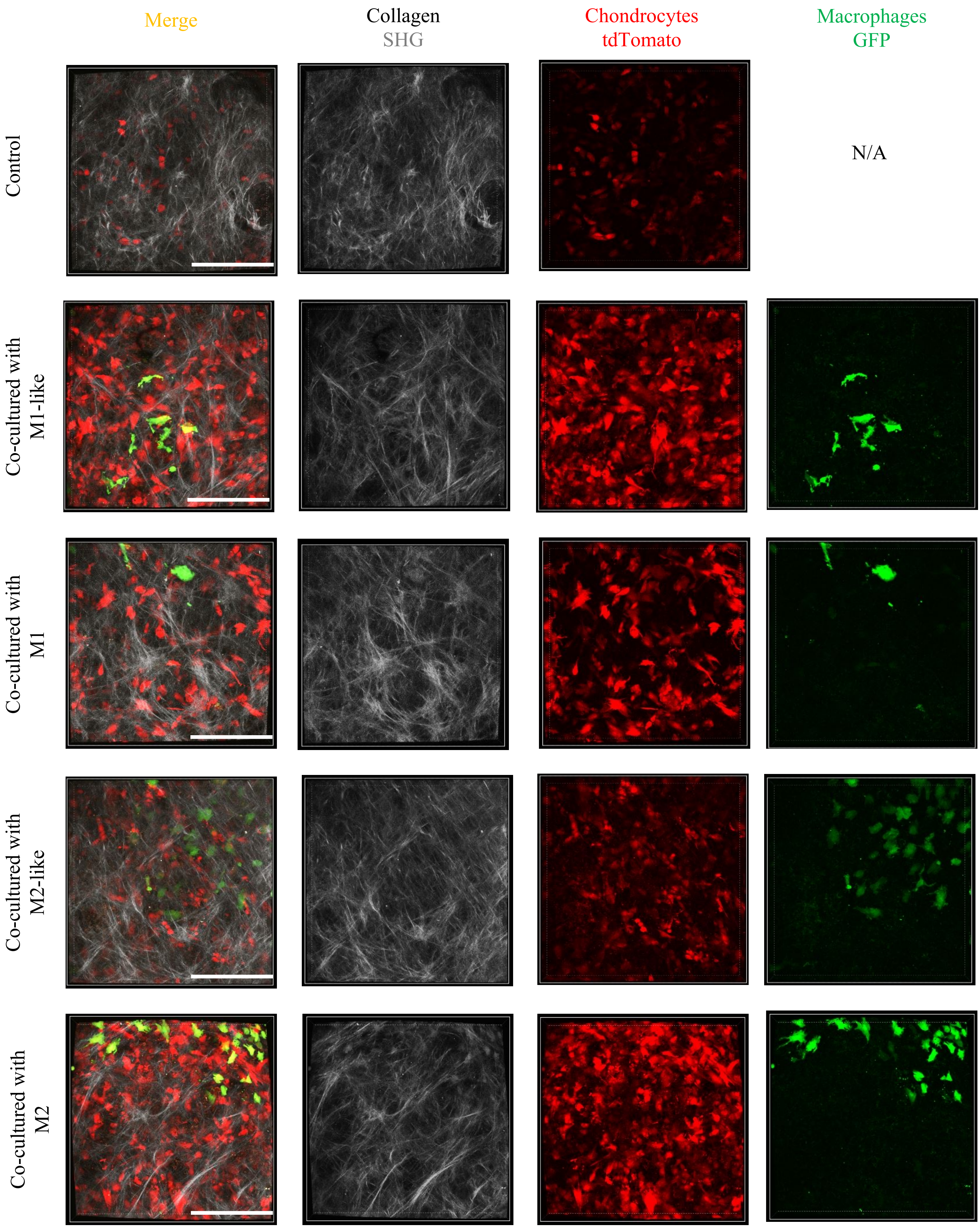

SI. 2b.

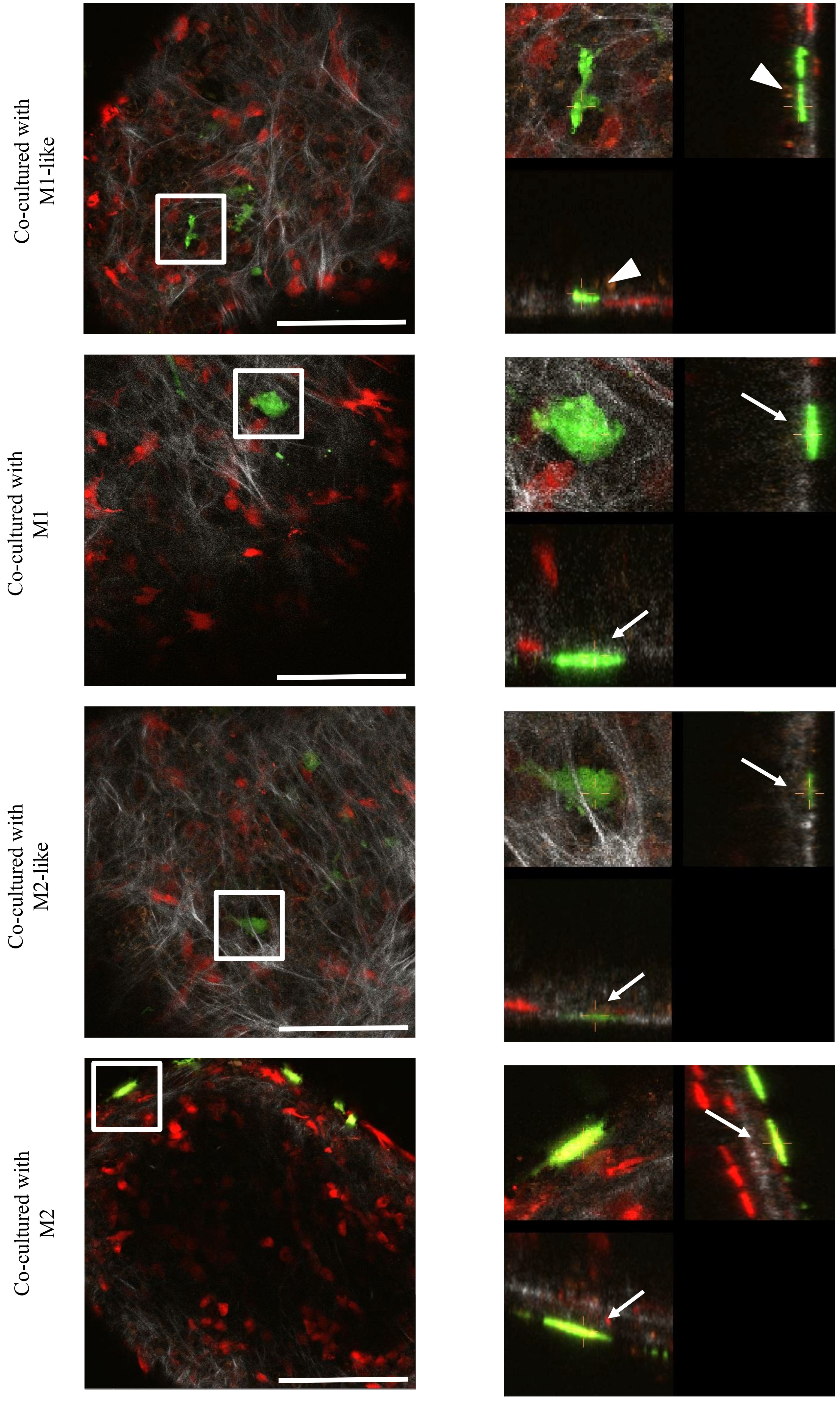
Co-cultured with M1-like
Co-cultured with M1
Co-cultured with M2-like
Co-cultured with M2

Supplementary Figure. 2. (SI. 2.)

Two-photon microscopy findings of cartilage pellets on day 14 of co-culture. White, red, green indicates collagen (second harmonic generation: SHG), chondrocytes (tdTomato), and macrophages (GFP), respectively. Three independent experiments were carried out and a representative experiment is shown.

SI .2a. 3D-reconstructed two-photon microscopy findings (XY axis). Scale bars: 200 μm.
SI. 2b. Two-photon microscopy findings (XY, YZ, and XZ axis). Scale bars: 200 μm.
Left: XY-axis of two photon images.
Right: Enlarged image of white squares from the image on the left image (XY, YZ, XZ axis). Brightness and contrast of images in the right panels were adjusted using the same parameters to facilitate their visibility. White arrowhead indicates macrophage in the pellet, and arrow indicates macrophage on the surface of the pellet.

Supplementary Table. 1. Primary antibodies for immunohistochemistry (SI. 3.)

Primary antibody	Supplier	Product	Working dilution for IHC	Antigen retrieval
Collagen type 1	LSL	Rabbit polyclonal	1:100	0.1% trypsin in PBS
Collagen type 2	LSL	Rabbit polyclonal	1:1000	2.5% hyaluronidase in PBS
Aggrecan	Proteintech	Rabbit polyclonal	1:200	Microwave<br>Citrate buffer (pH 6)
IL-1β	Abcam	Rabbit polyclonal	1:500	0.1% trypsin in PBS
IL-1RA	Abcam	Rabbit polyclonal	1:500	Microwave<br>Citrate buffer (pH 6)
IL-10	Proteintech	Rabbit polyclonal	1:200	Microwave<br>Citrate buffer (pH 6)

Supplementary Table. 2. Primary antibodies for immunofluorescence (SI. 4.)

Primary antibody	Supplier	Product	Working dilution for IF	Antigen retrieval
CD80	R&D Systems	Rat monoclonal	1:200	Microwave<br>Citrate buffer (pH 6)
CD206	R&D Systems	Goat polyclonal	1:200	Microwave<br>Citrate buffer (pH 6)

Supplementary Table. 3. Secondary antibodies for immunofluorescence (SI. 5.)

Secondary antibody	Supplier	Product	Working dilution for IF
Alexa Fluor 488	Abcam	Anti-goat	1:500
Alexa Fluor 647	Abcam	Anti-rat	1:500

Supplementary Table. 4. Primers for RT-PCR (SI. 6.)

Gene		Primer sequences
Collagen type 1	Froward	5′- ACG CCA TCA AGG TCT ACT GC -3′
	Reverse	5′- ACT CGA ACG GGA ATC CAT CG -3′
Collagen type 2	Froward	5′- ATC TGT GAA GAC CCA GAC TGC -3′
	Reverse	5′- CTC TGG GTC CTT GTT CAC CTG -3′
Aggrecan	Froward	5′- ACC ATC ACA GAG TCC GAG TG -3′
	Reverse	5′- ATT GCT CCT GGT CTG CAA CG -3′
CD80	Froward	5′- GCC TTG CCG TTA CAA CTC -3′
	Reverse	5′- TAC TCG GGC CAC ACT TTT -3′
CD206	Froward	5′- CAA GCG ATG TGC CTA CC -3′
	Reverse	5′- AAT GCT GTG GAT ACT TGC C -3′
IL-1β	Froward	5′- TGC CAC CTT TTG ACA GTG ATG -3′
	Reverse	5′- GTG CTG CTG CGA GAT TTG AA -3′
IL-6	Froward	5′- CTG CAA GAG ACT TCC ATC CAG -3′
	Reverse	5′- AGT GGT ATA GAC AGG TCT GTT GG -3′
IL-12	Froward	5′- AGC AGT AGC AGT TCC CCT GA -3′
	Reverse	5′- AGT CCC TTT GGT CCA GTG TG -3′
TNF-α	Froward	5′- CAG GCG GTG CCT ATG TCT C -3′
	Reverse	5′- CGA TCA CCC CGA AGT TCA GTA G -3′
IL-10	Froward	5′- GCT CTT ACT GAC TGG CAT GAG -3′
	Reverse	5′- CGC AGC TCT AGG AGC ATG TG -3′
IL-1RA	Froward	5′- CTC TGG AGT GAG ACG TTG GA -3′
	Reverse	5′- GGT TAG TAT CCC AGA TTC TGA AGG -3′
TGF-β	Froward	5′- CCA CCT GCA AGA CCA TCG AC -3′
	Reverse	5′- CTG GCG AGC CTT AGT TTG GAC -3′
MMP3	Froward	5′- GTC CTC CAC AGA CTT GTC CC -3′
	Reverse	5′- ATG CTG TGG GAG TTC CAT AGA G -3′
MMP13	Froward	5′- GAC AAG CAG TTC CAA AGG CTA C -3′
	Reverse	5′- ATG GGA AAC ATC AGG GCT CC -3′
ADAMTS5	Froward	5′- TGG CAG CAC CAA CAT AAC CA -3′
	Reverse	5′- ATG CCC ACA TAA ATC CTC TCG G -3′
GAPDH	Froward	5′- AGG TCG GTG TGA ACG GAT TTG -3′
	Reverse	5′- GGG GTC GTT GAT GGC AAC A -3′

Supplementary Table. 5. Secondary antibodies for immunofluorescence (SI. 7.)

Secondary antibody	Supplier	Product	Working dilution for IF
Alexa Fluor 488	Abcam	Anti-goat	1:500
Alexa Fluor 594	Abcam	Anti-rat	1:1000
